# Exploring the Relationship Between Religion, Health, and Sociodemographic Factors in Brazil During the COVID-19 Pandemic

**DOI:** 10.1007/s10943-026-02657-8

**Published:** 2026-04-10

**Authors:** Michael Ruberson Ribeiro da Silva, Patrícia Silva Bazoni, Ronaldo José Faria, Alciéllen Mendes da Silva, Eduardo Frizzera Meira, Jéssica Barreto Ribeiro dos Santos

**Affiliations:** Department of Pharmacy and Nutrition, Federal University of Espírito Santo, Alto Universitário, S/N, Guararema, Alegre, ES Brazil

**Keywords:** Religion, Health status, COVID-19, Epidemiology

## Abstract

This cross-sectional study examined the relationship between religion, sociodemographic factors, and health in Alegre, Brazil, during the COVID-19 pandemic. A total of 694 individuals were interviewed, of whom 49.6% (*n* = 344) were Catholic, 36.5% (*n* = 253) Evangelical/Protestant, 7.5% (*n* = 52) non-religious, and 6.5% (*n* = 45) affiliated with other religions. Logistic regression revealed factors linked to Catholicism (older age, marriage, medical appointments) and Protestantism (older age, mixed race, outlying residence, marriage, alcohol abstinence). Non-religious individuals were younger and, along with Catholics, had higher alcohol consumption, which may harm health. Protestants exhibited greater COVID-19 vaccine hesitancy. These results provide crucial insights into how religion intersects with public health during crises.

**Supplementary Information:**

The online version contains supplementary material available at 10.1007/s10943-026-02657-8.

## Introduction

Religious and spiritual practices are essential in shaping human behavior and well-being, impacting individual and community life (Bożek et al., 2020). Spirituality is defined as the personal pursuit of meaning and connection to a higher power or more significant purpose. Religion is seen as an organized system of beliefs and practices and offers frameworks that help individuals navigate existential issues, cope with adversity, and establish ethical guidelines (de Brito Sena et al., 2021; Paul Victor & Treschuk, 2020). Research over the last decade has shown that these practices significantly contribute to mental, emotional, and physical well-being. For instance, individuals who regularly engage in religious or spiritual practices report lower levels of psychological distress, including anxiety and depression, and experience greater life satisfaction and emotional stability (Bożek et al., 2020; Tirgari et al., 2022). These practices also foster resilience by providing meaning during crises, such as illness or grief (Manning et al., 2019; Roberto et al., 2020).

Besides their psychological benefits, religious and spiritual beliefs often promote moral and ethical behaviors integral to social cohesion. Values such as empathy, altruism, and justice, frequently emphasized in religious teachings, positively influence social interactions and contribute to developing supportive communities (Kimani, 2024). These communities offer a sense of belonging, essential for emotional support, and mitigate feelings of loneliness and social isolation, both rising concerns in modern society (Michaels et al., 2022).

Moreover, recent studies have shown a clear association between religious engagement and healthier lifestyles. Individuals engaged in religious activities tend to adopt behaviors conducive to physical health, such as reduced substance abuse, healthier diets, and more regular physical activity, leading to better overall health outcomes and longer life expectancy (Borges et al., 2021).

In Brazil, religious beliefs significantly shape personal decisions. A national survey revealed that a substantial portion of Brazilians believe their religious affiliations strongly influence decisions related to drug use, acceptance of medical advice, and smoking cessation (Borges et al., 2021).

Religious and spiritual practices have far-reaching effects on health, social cohesion, and moral development, and it is essential to understand their multifaceted impact on human life. Exploring this connection provides communities and health professionals with a holistic perspective on well-being, acknowledging the spiritual dimensions that contribute to individual health and resilience. This article evaluated the relationship between religion and health and its societal implications. It will provide a comprehensive understanding of how these dimensions relate to individuals’ and communities’ overall well-being and behavior during the COVID-19 pandemic.

## Methods

We conducted a cross-sectional study in Alegre, southeastern Brazil, to investigate the relationship between sociodemographic and health status and religious affiliation during the COVID-19 pandemic. We calculated a required sample size of 624 participants (Brazilian Institute of Geography & Statistics, 2010) to ensure a representative sample of 21,512 urban residents. The required sample size was calculated assuming an estimated prevalence of 50%, a 95% confidence level, and a design effect of 1.5 (Charan & Biswas, 2013). This conservative assumption was adopted because the survey was part of a broader project evaluating multiple COVID-19–related health and social outcomes; therefore, no single primary outcome prevalence was established a priori. Using a 50% prevalence is standard in cross-sectional studies when the true prevalence is unknown or when multiple indicators are assessed, because it corresponds to the maximum-variance scenario for a proportion and yields the largest required sample size, ensuring adequate precision across outcomes (Ranganathan et al., 2024).

We employed a probabilistic sampling technique proportional to the size of different areas within the municipality and randomly selected 10 out of 37 urban census tracts in the first stage. In the second stage, we aimed to interview an equal number of individuals from each tract, which reduced the likelihood of oversampling individuals in larger urban areas (Centers for Disease Control & Prevention, 2022). This approach ensured that our sample accurately represented the diverse regions of the municipality.

Data were collected through an on-site household survey conducted in November and December 2021. Trained field researchers administered a structured questionnaire during face-to-face interviews. The questionnaire consisted of four main sections: (1) sociodemographic characteristics (e.g., age, gender, education level, and income), (2) general health status (e.g., self-reported chronic conditions, medication use, and COVID-19 infection history), (3) lifestyle habits (e.g., smoking, alcohol consumption, and physical activity), and (4) religious status (e.g., catholic, evangelical/protestant, spiritualist, other religions, and without religion) (Supplementary Material). The questionnaire was pilot-tested before deployment to ensure clarity and reliability.

For data analysis, we first performed a descriptive analysis to summarize categorical variables as frequencies and percentages and continuous variables (e.g., age) as means and standard deviations. We then categorized participants based on their religious status (Catholic, Evangelical or Protestant, other religions, and no religious affiliation). Religious affiliation was classified according to the categories defined by the Brazilian Institute of Geography and Statistics (IBGE, 2025). Participants were asked to identify as 'Evangelical' or 'Protestant' without further distinction, as these terms are widely treated as interchangeable in the Brazilian social and statistical context. It is important to note that while IBGE questionnaires collect specific data on various denominations during the survey stage, official primary reports often present these results in an aggregated format to maintain longitudinal consistency and facilitate demographic comparisons. Consequently, this study adopted the overarching 'Protestant' category to align with national reporting standards and ensure comparability with official census trends (IBGE, 2025; da Silva & dos Santos, 2025). Henceforth, the group will be referred to solely as 'Protestant,' as this category historically encompasses the Evangelical tradition.

We then performed bivariate analyses to explore associations between sociodemographic, health, and lifestyle variables with religious status. We adopted logistic regression models to identify factors independently associated with religious status. Following standard model-building procedures, we included variables with a p-value of less than 0.20 in the bivariate analyses. The final multivariable model retained only those variables with a p-value of less than 0.05, indicating statistical significance. We reported odds ratios (OR) with 95% confidence intervals (CI) for each associated factor. The statistical analyses were conducted using Jamovi version 2.2.5 and Stata version 16.1.

The Research Ethics Committee (CEP) of the Federal University of Espírito Santo (UFES) approved this study under Opinion N°4.732.878. The informed consent form (ICF) was duly approved and signed by all interviewees. All stages of the study followed the ethical standards established by the human research committee and the principles of the 1964 Declaration of Helsinki, revised in 2013 (World Medical Association, 2013).

## Results

### Sociodemographic Characteristics

A total of 694 respondents were included in the study, divided into three main groups for analysis: Catholics (49.6%, *n* = 344), Protestants (36.5%, *n* = 253), and those with no religious affiliation (7.5%, *n* = 52). The “Other religions” group (6.5%, *n* = 45) was excluded from the multivariate analysis due to heterogeneity and small subgroup sizes. This category was predominantly composed of Spiritists (5.8%, n = 40), followed by a smaller segment (0.7%, *n* = 5) identifying with Afro-Brazilian religions or other minority groups.

Sociodemographic analysis revealed that Catholics were older than non-religious individuals (56.5 vs. 37.9 years, *p* < 0.001), and Protestants were older than non-religious (52.2 vs. 37.9 years, *p* < 0.001). Catholics and Protestants were more likely to be married than those without religion (47.1% and 48.0% vs. 9.6%, *p* < 0.001). Education showed that the non-religious had higher high school completion rates compared to Catholics and Protestants (*p* < 0.001). We observed income disparities, with a higher proportion of non-religious individuals earning ≤ 1 minimum wage than Catholics (*p* = 0.003) and Protestants (*p* = 0.001). Catholics were more likely to be white, while Protestants had a higher proportion of individuals identifying as mixed race or Black (*p* < 0.001) (Table 1).

**Table 1:** Sociodemographic characteristics of the study sample

Variables	No religion	Catholics	Protestants	Total	Differences
Age in years (mean. SD)	37.9 (16.6)	56.5 (18.5)	52.2 (18.2)	53.4 (18.9)	a. b. c
Sex					c
Female (n. %)	37 (71.2)	237 (68.9)	200 (79.1)	474 (73.0)	
Male (n. %)	15 (28.8)	107 (31.1)	53 (20.9)	175 (27.0)	
Race					c
White/caucasian (n. %)	28 (53.8)	195 (56.7)	95 (37.8)	318 (49.1)	
Mixed race (n. %)	15 (28.8)	104 (30.2)	104 (41.4)	223 (34.5)	
Black (n. %)	9 (17.3)	45 (13.1)	52 (20.7)	106 (16.4)	
Region of residence					b
Outlying districts (n. %)	21 (40.4)	108 (31.5)	67 (26.5)	196 (30.2)	
Municipal seat (n. %)	31 (59.6)	235 (68.5)	186 (73.5)	452 (69.8)	
Marital status					a. b
Single (n. %)	28 (53.8)	82 (23.8)	59 (23.4)	169 (26.1)	
Married (n. %)	5 (9.6)	162 (47.1)	121 (48.0)	288 (44.4)	
Others (n. %)	19 (36.5)	100 (29.1)	72 (28.6)	191 (29.5)	
Schooling					a. b. c
Some Elementary School Education (n. %)	8 (15.4)	139 (40.4)	95 (37.5)	242 (37.3)	
High School Graduate (n. %)	36 (69.2)	153 (44.5)	134 (53.0)	323 (49.8)	
Completed Technical or Higher Education (n. %)	8 (15.4)	52 (15.1)	24 (9.5)	84 (12.6)	
Income					a. c
≤ 1 minimum wage (n. %)	32 (64.0)	126 (38.8)	124 (52.1)	282 (46.0)	
1–2 minimum wages (n. %)	14 (28.0)	152 (46.8)	97 (40.8)	263 (42.9)	
> 2 minimum wages (n. %)	4 (8.0)	47 (14.5)	17 (7.1)	98 (11.1)	

SD: Standard Deviation; n: number of interviewees per variable to the total number of interviewees; %: percentage of the variable to the total number of respondents

a = statistically significant difference between non-religious and Catholics (*p*-value ≤ 0.05)

b = statistically significant difference between non-religious and Protestants (*p*-value ≤ 0.05)

c = statistically significant difference between Catholics and Protestants (*p*-value ≤ 0.05)

d = no difference among groups (*p*-value > 0.05)

### Clinical Characteristics

Catholics reported a higher frequency of medical appointments in the last year (81.3%) than non-religious individuals (65.4%) (*p* = 0.008), and private health insurance coverage was more common among Catholics (26.7%) than the non-religious (11.5%) (*p* = 0.018). Self-medication was significantly more frequent in the non-religious group (84.8%) than Catholics (67.9%) (*p* = 0.019). Alcohol consumption was also significantly higher among the non-religious (57.7%) than Catholics (28.7%) (*p* < 0.001). Protestants similarly reported more appointments (80.9%) than non-religious (65.4%) (*p* = 0.014) and showed a significantly lower rate of alcohol consumption (13.8% vs. 57.7%, *p* < 0.001). No differences were found between the groups’ quality of life or self-perceived health (Table 2).

**Table 2:** Clinical characteristics of the study sample

Variables	No religion	Catholics	Protestants	Total	Differences
Quality of life (mean. SD)	0.848 (0.162)	0.816 (0.197)	0.823 (0.194)	0.821 (0.193)	d
Health self-perception					d
Very good or good (n. %)	28 (53.8)	188 (54.7)	128 (50.6)	344 (53.0)	
Regular (n. %)	21 (40.4)	129 (37.5)	106 (41.9)	256 (39.4)	
Bad or Very Bad (n.%)	3 (5.8)	27 (7.8)	19 (7.5)	49 (7.6)	
Medical appointments in the last year					a.b
Yes (n. %)	34 (65.4)	279 (81.3)	203 (80.9)	516 (79.9)	
No (n. %)	18 (34.6)	64 (18.7)	48 (19.1)	130 (20.1)	
Physical activity					d
Yes (n. %)	18 (34.6)	125 (36.4)	92 (36.4)	235 (36.3)	
No (n. %)	34 (65.4)	218 (63.6)	161 (63.6)	413 (63.7)	
Private health plan					a
Yes (n. %)	6 (11.5)	92 (26.7)	54 (21.3)	152 (23.4)	
No (n. %)	46 (88.5)	252 (73.3)	199 (78.7)	497 (76.6)	
Self-medication					a. b
Yes (n. %)	39 (84.8)	224 (67.9)	163 (69.1)	426 (69.6)	
No (n. %)	7 (15.2)	106 (32.1)	73 (30.9)	186 (30.4)	
Polypharmacy					a
Yes (n. %)	6 (11.5)	81 (23.6)	47 (18.7)	134 (20.7)	
No (n. %)	46 (88.5)	262 (76.4)	205 (81.3)	513 (79.3)	
Use medicinal plants					d
Yes (n. %)	15 (29.4)	130 (38.8)	104 (41.8)	249 (39.2)	
No (n. %)	36 (70.6)	205 (61.2)	145 (58.2)	386 (60.8)	
Alcohol consumption					a. b. c
Yes (n. %)	30 (57.7)	98 (28.7)	35 (13.8)	163 (25.2)	
No (n. %)	22 (42.3)	244 (71.3)	218 (86.2)	484 (74.8)	
Smoke					b
Yes (n. %)	10 (19.2)	43 (12.5)	22 (8.7)	75 (11.6)	
No (n. %)	42 (80.8)	300 (87.5)	231 (91.3)	573 (88.4)	
Sleep					d
< 6 h (n. %)	11 (21.2)	79 (23.0)	61 (24.1)	151 (23.3)	
From 6 to 7 h (n. %)	14 (26.9)	92 (26.8)	72 (28.5)	178 (27.5)	
From 7 to 8 h (n. %)	17 (32.7)	108 (31.5)	86 (34.0)	211 (32.6)	
> 8 h (n. %)	10 (19.2)	64 (18.7)	34 (13.4)	108 (16.7)	
Diagnosis of COVID-19					d
Yes (n. %)	12 (23.1)	58 (16.9)	55 (22.1)	125 (19.4)	
No (n. %)	40 (76.9)	285 (83.1)	194 (77.9)	519 (80.6)	
Vaccinated against COVID-19					c
Yes (n. %)	51 (100.0)	340 (98.8)	242 (95.7)	633 (97.7)	
No (n. %)	0 (0.0)	4 (1.2)	11 (4.3)	15 (2.3)	

SD: Standard Deviation; n: number of interviewees per variable to the total number of interviewees; %: percentage of the variable to the total number of respondents. Polypharmacy was defined as 5 or more drugs in use

a = statistically significant difference between non-religious and Catholics (*p*-value ≤ 0,05)

b = statistically significant difference between non-religious and Protestants (*p*-value ≤ 0,05)

c = statistically significant difference between Catholics and Protestants (*p*-value ≤ 0,05)

d = no difference among groups (*p*-value > 0.05)

The most prevalent diseases were anxiety (44.2%), hypertension (45.5%), depression (19.9%), obesity (16.5%), and dyslipidemia (25.6%). Anxiety, depression, obesity, dyslipidemia, and other diseases showed no significant differences between religious groups. However, hypertension was significantly more common among Catholics (46.5%) and Protestants (48.6%) than those without religion (23.1%). Heart disease was significantly more common among Catholics (11.4%) and Protestants (11.5%) than those without religion (0.0%) (Table 3).

**Table 3:** Main diseases of the study sample

Variables	No religion	Catholics	Protestants	Total	Differences
Anxiety					d
Yes (n. %)	26 (50.0)	161 (46.8)	100 (39.5)	287 (44.2)	
No (n. %)	26 (50.0)	183 (53.2)	153 (60.5)	362 (55.8)	
Hypertension					a. b
Yes (n. %)	12 (23.1)	160 (46.5)	123 (48.6)	295 (45.5)	
No (n. %)	40 (76.9)	184 (53.5)	130 (51.4)	354 (54.5)	
Depression					d
Yes (n. %)	13 (25.0)	73 (21.2)	43 (17.0)	129 (19.9)	
No (n. %)	39 (75.0)	271 (78.8)	210 (83.0)	520 (80.1)	
Obesity					d
Yes (n. %)	7 (13.5)	55 (16.0)	45 (17.8)	107 (16.5)	
No (n. %)	45 (86.5)	289 (84.0)	208 (82.2)	542 (83.5)	
Dyslipidemia					d
Yes (n. %)	8 (15.4)	97 (28.2)	61 (24.1)	166 (25.6)	
No (n. %)	44 (84.6)	247 (71.8)	192 (75.9)	483 (74.4)	
Gastroesophageal reflux					d
Yes (n. %)	7 (13.5)	57 (16.6)	28 (11.1)	92 (14.2)	
No (n. %)	45 (86.5)	287 (83.4)	225 (88.9)	557 (85.8)	
Diabetes mellitus					d
Yes (n. %)	6 (11.5)	50 (14.5)	36 (14.2)	92 (14.2)	
No (n. %)	46 (88.5)	294 (85.5)	217 (85.8)	557 (85.8)	
Kidney diseases					d
Yes (n. %)	4 (7.7)	41 (11.9)	25 (9.9)	70 (10.8)	
No (n. %)	48 (92.3)	303 (88.1)	228 (90.1)	579 (89.2)	
Heart disease					a. b
Yes (n. %)	0 (0.0)	39 (11.4)	29 (11.5)	68 (10.5)	
No (n. %)	52 (100)	304 (86.6)	224 (88.5)	580 (89.5)	
Hypothyroidism					d
Yes (n. %)	3 (5.8)	34 (9.9)	18 (7.1)	55 (8.5)	
No (n. %)	49 (94.2)	309 (90.1)	235 (92.9)	593 (91.5)	
Asthma					d
Yes (n. %)	6 (11.5)	18 (5.2)	13 (5.1)	37 (5.7)	
No (n. %)	46 (88.5)	326 (94.8)	240 (94.9)	612 (94.3)	
Cancer					d
Yes (n. %)	0 (0.0)	17 (4.9)	6 (2.4)	23 (3.5)	
No (n. %)	52 (100.0)	327 (95.1)	247 (97.6)	626 (96.5)	

a = statistically significant difference between non-religious and Catholics (*p*-value ≤ 0.05)

b = statistically significant difference between non-religious and Protestants (*p*-value ≤ 0.05)

c = statistically significant difference between Catholics and Protestants (*p*-value ≤ 0.05)

d = no difference among groups (*p*-value > 0.05)

### Associated Factors to Religion

The multivariate analysis revealed several factors associated with religious affiliation. Age was a significantly associated factor for Catholics, with each additional year increasing the odds of being Catholic by 5% (OR = 1.05; 95% CI: 1.03–1.07; *p* < 0.001). Marital status also played a role, as married individuals were more likely to be Catholic than single individuals (OR = 4.68; 95% CI: 1.61–13.61; *p* =  0.005). Having medical appointments in the past year was associated with higher odds of being Catholic (OR = 2.50; 95% CI: 1.22–5.13; *p* = 0.012) (Table 4).

**Table 4:** Multivariate analysis of associated factors to religious belief

*No religion versus Catholics*			
Variables	OR	CI 95%	*P*-Value
Age in years	1.05	(1.03 – 1.07)	< 0.001
*Marital status*			
Single	1		
Married	4.68	(1.61 – 13.61)	0.005
Others	0.67	(0.30 – 1.52)	0.343*
*Medical appointments*			
No	1		
Yes	2.50	(1.22 – 5.13)	0.012

*No religion versus Protestants*			
Variables	OR	CI 95%	*P*-Value
Age in years	1.04	(1.02 – 1.07)	0.001
*Race*			
White/caucasian	1		
Mixed race	2.82	(1.21 – 6.57)	0.016
Black	2.48	(0.92 – 6.70)	0.072*
*Region of residence*			
Municipality seat	1		
Outlying districts	2.91	(1.33 – 6.45)	0.007
*Marital status*			
Single	1		
Married	4.49	(1.45 – 13.91)	0.009
Others	0.60	(0.23 – 1.54)	0.288*
*Alcohol consumption*			
Yes	1		
No	5.76	(2.62 – 12.66)	< 0.001

*Catholics versus Protestants*			
Variables	OR	CI 95%	*P*-Value
Age in years	0.98	(0.97 – 0.99)	< 0.001
Race			
White/caucasian	1		
Mixed race	1.89	(1.30 – 2.77)	< 0.001
Black	2.29	(1.41 – 3.73)	< 0.001
*Alcohol consumption*			
Yes	1		
No	3.26	(2.03 – 5.23)	< 0.001

OR: Odds Ratio; CI: Confidence Interval. *No statistical significance (p-value > 0.05)

For Protestants, age was positively correlated with Protestantism (OR = 1.04; 95% CI: 1.02–1.07; *p* = 0.001), indicating that older individuals were more likely to be Protestant. Race was also a significant factor, and individuals identifying as mixed race were more likely to be Protestant than whites (OR = 2.82; 95% CI: 1.21–6.57; *p* = 0.016). Living in outlying districts increased the likelihood of being Protestant (OR = 2.91; 95% CI: 1.33–6.45; p = 0.007), and being married was strongly associated with Protestantism (OR = 4.49; 95% CI: 1.45–13.91; *p* = 0.009). Alcohol abstention was also a strong predictor of Protestant affiliation (OR = 5.76; 95% CI: 2.62–12.66; *p* < 0.001).

Comparing Catholics and Protestants, age was inversely associated with Protestantism (OR = 0.98; 95% CI: 0.97–0.99; *p* < 0.001), and older individuals were less likely to be Protestant. Race remained significant, with mixed race (OR = 1.89; 95% CI: 1.30–2.77; *p* < 0.001) and Black (OR = 2.29; 95% CI: 1.41–3.73; *p* < 0.001) individuals more likely to be Protestant. Abstention from alcohol also strongly correlated with Protestantism (OR = 3.26; 95% CI: 2.03–5.23; *p* < 0.001), highlighting the behavioral differences between Catholics and Protestants (Table 4).

## Discussion

This study highlighted several sociodemographic and clinical differences between individuals of no religion, Catholics, and Protestants. These findings are consistent with established literature on the influence of religion on lifestyle and health. Rather than reflecting merely individual-level differences, these patterns may also express broader structural dynamics through which religious affiliation interacts with social organization, community networks, and institutional environments that shape health-related behaviors (da Silva & dos Santos, 2025; van der Hoek, 2025).

In our study, 49.6% of respondents identified as Catholic, 36.5% as Protestant, and 7.5% reported no religious affiliation. These results align with trends observed in Brazil’s national census data from 2000 and 2022. In 2000, Catholics comprised around 74% of the population, but by 2022, this had dropped to 56.7%, reflecting the decline we found in our findings. Meanwhile, the Protestant population has grown significantly, rising from 15.1% in 2000 to 26.9% in 2022, a trend mirrored in our data. The number of individuals with no religious affiliation remained steady, growing from 7.0% in 2000 to 9.3% in 2022, consistent with the 7.5% reported in our study. These shifts highlight the ongoing changes in Brazil’s religious landscape over the past two decades (da Silva & dos Santos, 2025). Recent analyses indicate that such transformations should not be interpreted solely as secularization, but rather as reconfigurations within a pluralizing religious field marked by institutional diversification, mobility between traditions, and the expansion of Pentecostal movements into new social and geographic spaces (da Silva & dos Santos, 2025; van der Hoek, 2025).

The tendency for those without religious affiliation to be younger reflects global patterns, where younger populations increasingly report no religious identity (da Silva et al., 2025; Pew Research Center, 2018). Pew Research Center’s global survey found that young adults in 41 out of 106 countries are significantly less likely to be affiliated with a religion. This generational shift toward secularism is often associated with higher educational attainment, which is similarly observed in this study, where the non-religious group had higher education levels than Catholics and Protestants (Pew Research Center, 2018). However, the category “no religion” is heterogeneous and may include individuals with spiritual beliefs or those disengaged from formal institutions rather than strictly secular individuals, reflecting processes of religious mobility and individualized belief systems documented in Brazil (da Silva & dos Santos, 2025; Monteiro de Barros et al., 2024).

In contrast, older individuals tend to hold stronger religious affiliations. This trend has been linked to factors such as increased spirituality and the role of religion in providing social support during aging (Malone & Dadswell, 2018). Some studies emphasize the importance of religiosity for older adults in preserving physical and mental health, which may explain the higher rates of medical appointments, medication use, and access to private health insurance among Catholics and Protestants (Zimmer et al., 2016). Importantly, age also represents a major epidemiological confounder when interpreting clinical outcomes. Because religious participants were significantly older, higher prevalence of chronic conditions likely reflects demographic composition rather than a direct effect of religiosity. This distinction is essential to avoid causal misinterpretation of associations observed in cross-sectional analyses (Fabbri et al., 2015; Nichols & Hayes-Larson, 2025).

The findings regarding the lower consumption of alcohol and smoking among Catholics and Protestants than non-religious individuals are supported by existing literature. Historically, Protestantism, especially evangelical branches, has emphasized temperance, leading to lower alcohol consumption (Chagas et al., 2023; Potter et al., 2014). The study by Lucchetti et al. (2014) further supports this, showing that higher religiousness correlates with stricter attitudes toward alcohol policies and fewer alcohol-related problems. Similarly, religious involvement has been consistently linked with lower smoking rates. Studies have found that religiously active individuals are less likely to smoke, which could be a result of religious teachings that promote health-conscious behaviors (Hussain et al., 2019; Koenig et al., 1998).

The data showing lower educational attainment among Protestants, particularly in Brazil, aligns with previous research indicating that evangelical Protestants tend to have lower education levels than Catholics or other religious groups. Peres et al. (2018) found that Protestants were more likely to belong to mixed-race or Black ethnic groups and had lower income levels than Catholics, who were more frequently white and had higher incomes. These sociodemographic factors may contribute to the observed disparities in education and income between religious groups and differences in access to healthcare resources. Such disparities should be interpreted within broader structural inequalities in Brazilian society, where religious institutions often operate within specific socioeconomic niches and may function as support systems in contexts of limited access to formal resources, particularly in urban peripheries and underserved regions (van der Hoek, 2025).

Another key finding is the variation in COVID-19 vaccination rates, where Protestants showed lower rates than Catholics, consistent with studies that have identified vaccine hesitancy among evangelical Christian groups. Guidry et al. (2022) suggest that the skepticism toward vaccines in some Protestant communities may stem from religious or political beliefs, contributing to lower vaccination rates. Evidence from a recent systematic review indicates that religious leaders can play a central role in shaping vaccine attitudes by influencing trust, risk perception, and acceptance of public health interventions (Ali Sheikhi et al., 2025).

In this regard, vaccine attitudes were shaped not only by theological considerations but also by the intersection of religious identity with political discourse, trust in institutions, and exposure to misinformation networks, indicating that such differences likely reflect sociopolitical dynamics rather than doctrinal positions alone (Corcoran et al., 2021; Jin et al., 2024). In the Brazilian pandemic context, this interplay may have been associated with phenomena described as “religious denialism,” in which public health guidance was contested or reframed through religious–political narratives, potentially reinforcing vaccine skepticism in specific communities.

A key limitation of this study is the use of a single aggregated “Protestant” category, which may obscure important internal diversity within the Brazilian religious field. In Brazil, the terms “Evangelical” and “Protestant” are often used interchangeably in surveys and public discourse. However, this broad group is highly heterogeneous, including mainline/mission traditions as well as Pentecostal and Neo-Pentecostal movements. Although we followed the IBGE convention of reporting aggregated categories, this approach limits our ability to detect subgroup-specific differences in sociodemographic profiles, health-related behaviors, and levels of institutional trust. For example, evidence suggests that Pentecostal expansion in urban peripheries is linked to distinctive social and spiritual functions that may shape health decisions differently from those observed in mainline Protestant groups (IBGE, 2025, da Silva & dos Santos, 2025; van der Hoek, 2025). Future studies should use disaggregated religious measures to capture these nuances and better examine how specific institutional and community dynamics relate to health outcomes and public health responses.

This study is also limited by its cross-sectional design, which did not allow for establishing causal relationships between sociodemographic and health status and religious affiliation. Additionally, the research was conducted in a single municipality, which may restrict the generalizability of the findings to other regions with different sociodemographic characteristics, or religious contexts. Despite these limitations, the study provides valuable insights into how religious affiliation may be related to social well-being and health during a public health crisis, such as the COVID-19 pandemic, particularly relevant in smaller communities, where social and religious ties tend to be stronger and more prominent.

These findings suggest that religion operates as a socially embedded context that can shape health behaviors and public health responses, particularly during crises such as the COVID-19 pandemic. Future research should utilize disaggregated religious measures and longitudinal designs to clarify subgroup-specific patterns and better distinguish compositional effects from potential mechanisms linking religion and health.

## Conclusion

This study’s findings align with the broader literature on the intersection of religion, sociodemographic characteristics, and health behaviors. The differences in alcohol consumption, smoking, education, and health access between Catholics, Protestants, and the non-religious highlight the complex ways in which religion influences lifestyle choices and clinical outcomes. While religion may offer certain health benefits through community support and health-conscious behaviors, disparities in education and income persist, particularly among Protestants, which may contribute to unequal health outcomes.

## Supplementary Information

Below is the link to the electronic supplementary material.

Supplementary file1 (PDF 615 KB)
