# Supplementary material for: Exploring the Relationship Between Religion, Health, and Sociodemographic Factors in Brazil During the COVID-19 Pandemic
Source: J Relig Health. 2026 Apr 10;65(4):4055–69. doi: 10.1007/s10943-026-02657-8 (PMC13481583; doi:10.1007/s10943-026-02657-8)
Supplement: Supplementary file 1 [file 10943_2026_2657_MOESM1_ESM.pdf]

SUPPLEMENTARY MATERIAL – DATA COLLECTION QUESTIONNAIRE

PART A - SOCIODEMOGRAPHIC DATA

Name:

Email: | **Telephone:**

Neighborhood/District: | **Mother's name:**

City of origin: | **Questionnaire number:**

1- Sex: 1. Female 2. Male |____|

2- Date of birth: ______/______/______or __________years

3- What is your weight?
_________ Kilograms |____|

4- How tall are you?
_________ centimeters or _________ metre |____|

5- Race or color: |____|
1. White
2. Black
3. Brown
4. Yellow
5. Indigenous
6. Albino
7. Other:
Specify:________________________

6- Marital status: |____|
1. Single
2. Married
3. Stable Union
4. Divorced
5. Separate
6. Widower
7. Other
Specify:________________________

7- Education: _________full years of study |____|
1. No schooling
2. Incomplete elementary school (1st grade)
3. Complete elementary school (1st grade)
4. Incomplete high school (2nd degree)
5. Complete high school (2nd degree)
6. Complete technician
7. Complete Superior
8. Complete Master's Degree
9. Full PhD

8- You live in: |____|
1. Own house or apartment (of family or friends)
2. Rented house or apartment
3. Bedroom, room, or kitchenette rented or borrowed
4. Republic
If another, (hotel, asylum, etc.) specifies: _____________________________________

9- Religion/Belief |____|
1. No religion
2. Catholic
3. Evangelical/Protestant
4. Spiritist
5. Other
Specify: ____

10- Do you live temporarily in Alegre for study or work? |____|
1. Yes, study 2. Yes, work 3. No

11- Lives with |____|
1. Alone
2. Family members (Parents and/or
33. Relatives (grandparents/uncles)
44. Friends

12- Occupation |____|
1. Self-employed/Professional/Own account
2. Employer/Entrepreneur /Entrepreneur
3. Formal private employee (CLT or contract)
4. Civil servant (Public companies, e.g. bank of Brazil, cashier, etc.)
5. Public servant
6. Student
7. Other. Specify:____

13- What is your profession? ____

**14- Gross monthly income in minimum wages:**<br>1. ≤ 1 Salary (< R$ 1,100)<br>2. 1 to 2 Salaries (R$ 1.101 - R$ 2.200)<br>3. 2 to 4 Salaries (R$ 2.201 - R$ 4.400)<br>4. 4 to 10 salaries (R$ 4.401 - R$ 11.000)<br>5. > 10 Salaries (> R$ 11,001)	|____|
**PART B - GENERAL HEALTH AND USE OF HEALTH SERVICES**	
**15- Generally speaking , you consider your own state of health as:**<br>1. Very good 3. Regular 5. Too bad<br>2. Good 4. Bad	|____|
**16- In the last 15 days, you have stopped doing some of your usual activities (e.g. leaving home, going for a walk or working) for any health problems?**<br>1. Yes 2. No	|____|
**17- In the last 15 days, have you been bedridden for any health problems?**<br>1. Yes 2. No	|____|
**18- From 1 year to now, how many times have you consulted with a doctor?**<br>0 1 2 3 4 5 >5	|____|
**19- From 1 year to now, how many times have you consulted with a dentist?**<br>0 1 2 3 4 5 >5	|____|
**20- From 1 year to now, how many times have you consulted with a nutritionist?**<br>0 1 2 3 4 5 >5	|____|
**21- From 1 year to now, how many times have you consulted with another professional?**<br>0 1 2 3 4 5 >5<br>Which:____	|____|
**22- From 1 year to now, how many times have you been admitted to a hospital? Consider hospitalization when you have slept in the hospital for at least one night for health problem or accident with you.**<br>1. Not once 2. Once 3. Two or more	|____|
**23- In addition to the care provided by SUS, do you have private health insurance?**<br>1. Yes 2. No	|____|
**24- Does your private health plan pay or provide medicines for you?**<br>1. Yes 2. No 3. You don't have private health insurance	|____|
**25- What is the main problem when you need health services?**<br>1. It's hard to get to the point of care (no transportation or away from home)<br>2. It's hard to make a doctor's appointment (have to stand in line)<br>3. The price of services is very expensive<br>4. It's hard to get a doctor who attends at the time you need it<br>5. Low quality of care<br>6. Lack of medicines and supplies<br>7. You don't have difficulties when you need medical services<br>8. You don't need medical services<br>9. If other, specify: ____________________________________	|____|
**Have you ever in your life a doctor or other health care professional said you have or have you had any of the following diseases? (If necessary, check more options.)**<br>1. Yes 2. No<br>**26- Asthma**<br>**27- Anxiety**<br>**28- Rheumatoid arthritis**<br>**29- Cancer / Tumor / Neoplasms**<br>**30- Depression**<br>**31- Diabetes Mellitus**<br>**32- Dyslipidemia (high cholesterol and /or triglycerides)**<br>**33- Heart diseases (heart attack, heart failure, angina, etc.)**	 <br><br>|____| |____| |____| |____| |____| |____| |____| |____|

**34- Gastroesophageal reflux disease**<br>**35- Kidney diseases of any nature**<br>**36- Hypertension (high blood pressure)**<br>**37- Obesity (overweight)**<br>**38- Hypothyroidism**<br>**39- Other, please specify:** ______________________________	|____| |____| |____| |____| |____| |____|
**40- Has a disability:**<br>1. Yes 2. No	|____|
**41- What type of disability**<br>1. Hearing 3. Cognitive 5. Other<br>2. Visual 4. Physics<br>**Especificar:** ______________________________	|____|
**PART C – COVID-19**	
**42- Got vaccinated?**<br>1. Yes 2. No	|____|
**43- Which vaccine?**<br>1. Coronavac 3. Pfizer 5. Other<br>2. Astra-zeneca 4. Jannsen	|____|
**44- How many doses?**<br>1. One serving 2. Two doses 3. Three doses 4. Single dose	|____|
**45- Have you had an allergic reaction to the vaccine?**<br>1. Yes 2. No	|____|
**46- If so, which one?**<br>Specify: ______________________________	|____|
**47- Did you have COVID-19?**<br>1. Yes 2. No	|____|
**48- When?**<br>1. Before vaccination 3. After the second dose<br>2. After the first dose	|____|
**49- Have you received any medication or treatment?**<br>1. Yes 2. No	|____|
**50- If yes, was it prescribed or indicated by any professional?**<br>1. Yes, medical prescription 4. Yes, referral from friends or relatives<br>2. Yes, pharmaceutical indication 5. No, you searched the internet<br>3. Yes, referral from another professional 6. No, he took it on his own	|____|
**51- If so, what medications were used?**<br>Specify: ______________________________	
**52- Were you hospitalized?**<br>1. Yes 2. No<br>Time in days: ______________________________	|____|
**PART D – USE OF HEALTH SERVICES**	
**53- 53- In the last year you used the Municipal Basic Pharmacy?**<br>1. Yes 2. No	|____|
**54- If yes, what is your level of satisfaction with the service offered?**<br>Totally Unsatisfied 0 ______________________________ 10 Totally Satisfied	|____|
**55- In the last year you used the Emergency Room / Municipal Emergency Room?**<br>1. Yes 2. No	|____|
**56- If so, what is your level of satisfaction with the service offered?**<br>Totally Unsatisfied 0 ______________________________ 10 Totally Satisfied	|____|

**57- In the last year you used the Municipal Medical Center?**<br>1. Yes 2. No	
**58- If so, what is your level of satisfaction with the service offered?**<br>Totally Unsatisfied 0 ________ 10 Totally Satisfied	
**59- In the last year did you use the Basic Health Unit?**<br>1. Yes 2. No<br>If yes, specify which: ________	
**60- If so, what is your level of satisfaction with the service offered?**<br>Totally Unsatisfied 0 ________ 10 Totally Satisfied	
**61- In the last year you used the Hospital of Alegre?**<br>1. Sim 2. Não	
**62- If so, what is your level of satisfaction with the service offered?**<br>Totally Unsatisfied 0 ________ 10 Totally Satisfied	
**63- In the last year you used the Immunization Center of Alegre?**<br>1. Sim 2. Não	
**64- If so, what is your level of satisfaction with the service offered?**<br>Totally Unsatisfied 0 ________ 10 Totally Satisfied	
**65- In the last year did you use the Psychosocial Care Center of Alegre?**<br>1. Sim 2. Não	
**66- If so, what is your level of satisfaction with the service offered?**<br>Totally Unsatisfied 0 ________ 10 Totally Satisfied	
**67- In the last year have you had any private medical consultation in Alegre?**<br>1. Sim 2. Não	
**68- If so, what is your level of satisfaction with the service offered?**<br>Totally Unsatisfied 0 ________ 10 Totally Satisfied	
**69- In the last year you used any Private Pharmacy in Alegre?**<br>1. Sim 2. Não	
**70- If so, what is your level of satisfaction with the service offered?**<br>Totally Unsatisfied 0 ________ 10 Totally Satisfied	
**PART E - USE OF MEDICINES**	
In the next questions, we want to know some information about the use of medications by you.<br>Note: If you do not use medicines, skip to question no. 121.	
**71- In the last 15 days, have you used medications?**<br>1. Yes. How many?_________ 2. No 99. NSA	
**72- Do you need help from someone else to take your medications?**<br>1. No 2. Yes, sometimes 3. Yes, always	
**73- How many dollars have you spent on medicines in the last 30 days?**<br>R$______,_____ reais<br>1. I didn't spend anything 2. I don't know the value	
**74- Do you usually use any medications on your own?**<br>1. Yes 2. No 99. NSA	
**75- In the last 15 days have you stopped using any prescription drugs?**<br>1. Yes 2. No	
**MEDICATION 1**	
**(copy this information preferably from the packaging or prescription)**	

**76- Name:**	
**77- Dosage:**	|___|
**78- Frequency of use:**	|___|
**79- How long have you been using this medicine?**<br>1. Less than 1 year 2. 1 year or more	|___|
**80- Where has this medicine been prescribed or recommended?**<br>1. In consultation with the doctor 4. On the radio/TV/newspaper<br>2. In consultation with the dentist 5. Friends or relatives or neighbors<br>3. In the pharmacy 6. Use on your own	|___|
**81- Where did you last get this medicine?**<br>1. In the SUS or public pharmacy 3. Other<br>2. In the commercial pharmacy<br>Specify: ____	|___|
**82- Do you have any problems getting or using this medicine?**	
1. Nope. No problem 4. Yes. There's no one to look for<br>2. Yes. Not in the SUS pharmacy 5. Yes. You don't have money to buy<br>3. Yes. I got sick while using the medicine 6. Yes. Another problem<br>Specify: ______________________	|___|
**83- Have you stopped taking this medication for some reason in the last 15 days?**<br>1. Yes 2. No	|___|
**84- Why did you stop taking this medicine in the last 15 days.**	|___|
**MEDICATION 2**	
**(copy this information preferably from the packaging or prescription)**	
**85- Name:**	|___|
**86- Dosage:**	|___|
**87- Frequency of use:**	|___|
**88- How long have you been using this medicine?**<br>1. Less than 1 year 2. 1 year or more	|___|
**89- Where has this medicine been prescribed or recommended?**<br>1. In consultation with the doctor 4. On the radio/TV/newspaper<br>2. In consultation with the dentist 5. Friends or relatives or neighbors<br>3. In the pharmacy 6. Use on your own	|___|
**90- Where did you last get this medicine?**<br>1. In the SUS or public pharmacy 3. Other<br>2. In the commercial pharmacy<br>Specify: ____	|___|
**91- Do you have any problems getting or using this medicine?**	
1. Nope. No problem 4. Yes. There's no one to look for<br>2. Yes. Not in the SUS pharmacy 5. Yes. You don't have money to buy<br>3. Yes. I got sick while using the medicine 6. Yes. Another problem<br>Specify: ______________________	|___|
**92- Have you stopped taking this medication for some reason in the last 15 days?**<br>1. Yes 2. No	|___|
**93- Why did you stop taking this medicine in the last 15 days.**	|___|
**MEDICATION 3**	

**(copy this information preferably from the packaging or prescription)**	
**94- Name:**	|___|
**95- Dosage:**	|___|
**96- Frequency of use:**	|___|
**97- How long have you been using this medicine?**<br>1. Less than 1 year 2. 1 year or more	|___|
**98- Where has this medicine been prescribed or recommended?**<br>1. In consultation with the doctor 4. On the radio/TV/newspaper<br>2. In consultation with the dentist 5. Friends or relatives or neighbors<br>3. In the pharmacy 6. Use on your own	|___|
**99- Where did you last get this medicine?**<br>1. In the SUS or public pharmacy 3. Other<br>2. In the commercial pharmacy<br>Specify: ____	|___|
**100- Do you have any problems getting or using this medicine?**	
1. Nope. No problem 4. Yes. There's no one to look for<br>2. Yes. Not in the SUS pharmacy 5. Yes. You don't have money to buy<br>3. Yes. I got sick while using the medicine 6. Yes. Another problem<br>Specify: _________________________	|___|
**101- Have you stopped taking this medication for some reason in the last 15 days?**<br>1. Yes 2. No	|___|
**102- Why did you stop taking this medicine in the last 15 days.**	|___|
**MEDICATION 4**	
**(copy this information preferably from the packaging or prescription)**	
**103- Name:**	|___|
**104- Dosage:**	|___|
**105- Frequency of use:**	|___|
**106- How long have you been using this medicine?**<br>1. Less than 1 year 2. 1 year or more	|___|
**107- Where has this medicine been prescribed or recommended?**<br>1. In consultation with the doctor 4. On the radio/TV/newspaper<br>2. In consultation with the dentist 5. Friends or relatives or neighbors<br>3. In the pharmacy 6. Use on your own	|___|
**108- Where did you last get this medicine?**<br>1. In the SUS or public pharmacy 3. Other<br>2. In the commercial pharmacy<br>Specify: ____	|___|
**109- Do you have any problems getting or using this medicine?**<br>1. Nope. No problem 4. Yes. There's no one to look for<br>2. Yes. Not in the SUS pharmacy 5. Yes. You don't have money to buy<br>3. Yes. I got sick while using the medicine 6. Yes. Another problem<br>Specify: _________________________	|___|
**110- Have you stopped taking this medication for some reason in the last 15 days?**<br>1. Yes 2. No	|___|
**111- Why did you stop taking this medicine in the last 15 days.**	|___|

MEDICATION 5	
**(copy this information preferably from the packaging or prescription)**	
**112- Name:**	|___|
**113- Dosage:**	|___|
**114- Frequency of use:**	|___|
**115- How long have you been using this medicine?**<br>1. Less than 1 year 2. 1 year or more	|___|
**116- Where has this medicine been prescribed or recommended?**<br>1. In consultation with the doctor 4. On the radio/TV/newspaper<br>2. In consultation with the dentist 5. Friends or relatives or neighbors<br>3. In the pharmacy 6. Use on your own	|___|
**117- Where did you last get this medicine?**<br>1. In the SUS or public pharmacy 3. Other<br>2. In the commercial pharmacy<br>Specify: ____________________________________	|___|
**118- Do you have any problems getting or using this medicine?**	
1. Nope. No problem 4. Yes. There's no one to look for<br>2. Yes. Not in the SUS pharmacy 5. Yes. You don't have money to buy<br>3. Yes. I got sick while using the medicine 6. Yes. Another problem<br>Specify: ________________________	|___|
**119- Have you stopped taking this medication for some reason in the last 15 days?**<br>1. Yes 2. No	|___|
**120- Why did you stop taking this medicine in the last 15 days.**	|___|
**PART F - USE OF TEAS AND MEDICINAL PLANTS**	
In the next questions, we want to know some information about the use of teas and medicinal plants.<br>Note: If you do not use teas or medicinal plants, skip to question no. 132.	
**121- Have you had any tea or used a medicinal plant in the last 15 days?**<br>1. Yes 2. No 99. NSA	|___|
**PLANT 1**	
**122- Name:** ____________________________________<br>**Indication:** ____________________________________	
**123- Part used:**<br>1. Leaf 3. Stalk 5. Flower<br>2. Root 4. Bark 6. Sachet 99. NSA	|___|
**PLANT 2**	
**124- Name:** ____________________________________<br>**Indication:** ____________________________________	|___|
**125- Part used:**<br>1. Leaf 3. Stalk 5. Flower<br>2. Root 4. Bark 6. Sachet 99. NSA	|___|
**PLANT 3**	
**126- Name:** ____________________________________<br>**Indication:** ____________________________________	
**127- Part used:**<br>1. Leaf 3. Stalk 5. Flower<br>2. Root 4. Bark 6. Sachet 99. NSA	|___|
**PLANT 4**	
**128- Name:** ____________________________________<br>**Indication:** ____________________________________	

**129- Part used:**<br>1. Leaf 3. Stalk 5. Flower<br>2. Root 4. Bark 6. Sachet 99. NSA	|___|
**PLANT 5**	
**130- Name:** ____<br>**Indication:** ____	
**131- Part used:**<br>1. Leaf 3. Stalk 5. Flower<br>2. Root 4. Bark 6. Sachet 99. NSA	|___|
**PART G - USE OF HOMEOPATHIC, HERBAL MEDICINES, MANIPULATED AND HOMEMADE**	
**132- Do you make use of homeopathic medicines?**<br>1. Yes 2. No<br>Indication: ____	|___|
**133- Does it make use of Bach's Floral?**<br>1. Yes 2. No<br>Indicação: ____	|___|
**134- Do you use herbal medicines?**<br>1. Yes 2. No<br>Indication: ____	|___|
**135- Do you use manipulated medications?**<br>1. Yes 2. No<br>Indication: ____	|___|
**136- Use of home medicines?**<br>1. Yes 2. No<br>Specify: ____<br>Indication: ____	|___|
**PART H - LIFESTYLE HABITS**	
**Think about your weekly routine: what are the meals you usually eat on the day?**<br>1. Yes 2. No<br>**137- Breakfast**<br>**138- Morning snack**<br>**139- Lunch**<br>**140- Snack or afternoon coffee**<br>**141- Dinner or evening coffee**<br>**142- LSnack before bed (supper)**	 <br>|___| |___| |___| |___| |___| |___|
**143- How often do you drink alcohol?**<br>1. I never drink 3. Weekly<br>2. Daily 4. Monthly	|___|
**144- You do REGULAR physical activity, that is, at least 30 minutes.**<br>1. Yes 2. No<br>Weekly frequency: ____	|___|
**145- Do you currently smoke?**<br>1. Yes 2. No	|___|
**146- How many hours a day do you usually sleep?**<br>1. < 6 hours 3. 7 to 8 hours<br>2. 6 to 7 hours 4. > 8 hours	|___|

QUESTIONNAIRE______________________________DATE:_____/___/____

INTERVIEWEE____

EQ-5D-3L

At this time, we want to understand how your current health status is. I will read three statements in each question and you will indicate which one best describes your state of health today.

38A- Mobility

- ❍ 1 - I have no problems in walking about
- ❍ 2 - I have some problems in walking about
- ❍ 3 - I am confined to bed

38B- Self-care

- ❍ 1 - I have no problems with self-care
- ❍ 2 - I have some problems washing or dressing myself
- ❍ 3 - I am unable to wash or dress myself

38C- Usual Activities (e.g. work, study, housework, family or leisure activities)

- ❍ 1 - I have no problems with performing my usual activities
- ❍ 2 - I have some problems with performing my usual activities
- ❍ 3 - I am unable to perform my usual activities

38D- Pain/Discomfort

- ❍ 1 - I have no pain or discomfort
- ❍ 2 - I have moderate pain or discomfort
- ❍ 3 - I have extreme pain or discomfort

38E- Anxiety/Depression

- ❍ 1- I am not anxious or depressed
- ❍ 2 - I am moderately anxious or depressed
- ❍ 3 - I am extremely anxious or depressed

VISUAL ANALOGIC SCALE

To help people say how good or bad their health status is, we have drawn a scale (similar to a thermometer) on which the best health status you can imagine is marked by 100 and the worst health status you can imagine is marked by 0.

We would like you to indicate on this scale how good or bad is, in your opinion, your state of health today. Please draw a line from the square below to the point on the scale that best describes your health status today.

The best health
you can imagine

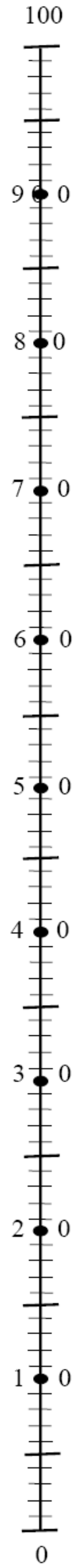

The worst health
you can imagine

WHAT IS YOUR HEALTH STATUS TODAY?

MAKE A LINE LEAVING THIS FRAME TO THE SCALE ON THE SIDE
